# Clinical *Aspergillus* Signatures in COPD and Bronchiectasis

**DOI:** 10.3390/jof8050480

**Published:** 2022-05-05

**Authors:** Pei Yee Tiew, Kai Xian Thng, Sanjay H. Chotirmall

**Affiliations:** 1 Department of Respiratory and Critical Care Medicine, Singapore General Hospital, Singapore 168753, Singapore; gmstpye@nus.edu.sg; 2 Duke-NUS Medical School, Singapore 169857, Singapore; 3 Lee Kong Chian School of Medicine, Nanyang Technological University, Singapore 308232, Singapore; kaixian001@e.ntu.edu.sg; 4 Department of Respiratory and Critical Care Medicine, Tan Tock Seng Hospital, Singapore 308433, Singapore

**Keywords:** mycobiome, bronchiectasis, COPD, fungi, *Aspergillus*, next-generation sequencing

## Abstract

Pulmonary mycoses remain a global threat, causing significant morbidity and mortality. Patients with airways disease, including COPD and bronchiectasis, are at increased risks of pulmonary mycoses and its associated complications. Frequent use of antibiotics and corticosteroids coupled with impaired host defenses predispose patients to fungal colonization and airway persistence, which are associated with negative clinical consequences. Notably, *Aspergillus* species remain the best-studied fungal pathogen and induce a broad spectrum of clinical manifestations in COPD and bronchiectasis ranging from colonization and sensitization to more invasive disease. Next-generation sequencing (NGS) has gained prominence in the field of respiratory infection, and in some cases is beginning to act as a viable alternative to traditional culture. NGS has revolutionized our understanding of airway microbiota and in particular fungi. In this context, it permits the identification of the previously unculturable, fungal composition, and dynamic change within microbial communities of the airway, including potential roles in chronic respiratory disease. Furthermore, inter-kingdom microbial interactions, including fungi, in conjunction with host immunity have recently been shown to have important clinical roles in COPD and bronchiectasis. In this review, we provide an overview of clinical *Aspergillus* signatures in COPD and bronchiectasis and cover the current advances in the understanding of the mycobiome in these disease states. The challenges and limitations of NGS will be addressed.

## 1. Introduction

Microbes play a central role in the pathogenesis and progression of chronic respiratory diseases (CRDs), including chronic obstructive pulmonary disease (COPD) and bronchiectasis [1,2,3,4]. While bacterial infection has been well-studied, the precise role of fungi remains uncertain [1,2,5,6]. Recent work by our group and others has brought about renewed interest and focus on this important knowledge gap, and demonstrates the important and underappreciated role of fungi and the mycobiome in COPD and bronchiectasis [5,7,8,9,10]. Dedicated research addressing the specific role of fungi in CRD has been highlighted as a research priority by major CRD registries and consortia, signaling global interest and the increasing emphasis on its importance as a key area of unmet need [11,12]. Fungi may lead to various presentations in patients with CRD. In the immune hyperreactive state, it induces sensitization responses, such as that seen in severe asthma with fungal sensitization (SAFS) or allergic bronchopulmonary mycoses (ABPM), resulting in high symptomatic burden and poor disease control [13]. With accompanying immune dysfunction, fungi may manifest acute, chronic, and/or invasive infection phenotypes that lead to high mortality [14,15]. The average person breathes 22,000 times a day, inhaling approximately 11,000 L of air. Fungal spores are abundant in outdoor air, and it is estimated that up to 50,000 fungal spores/m^3^ of air are inhaled daily [16,17,18]. While such constant inhalation and exposure to fungal spores pose no risk to healthy (immunocompetent) individuals, fungal-associated disease may develop in individuals with CRD, including COPD and bronchiectasis, of which the *Aspergillus*-species remain the best studied [19,20]. Patients with COPD and bronchiectasis remain predisposed to *Aspergillus*-associated conditions ranging from asymptomatic colonization to *Aspergillus* sensitization (AS) or *Aspergillus* bronchopulmonary aspergillosis (ABPA) in immune-hypersensitive patients. In those with relative or frank immunodeficiency, chronic and/or invasive pulmonary aspergillosis (CPA or IPA) may be seen, with the latter usually characterized by late diagnosis and high mortality [6,16,17]. Fungi represent a microbial kingdom comprising more than 1.5 million estimated species, with the majority being unculturable using standard laboratory media [21,22]. Therefore, the use of next-generation sequencing (NGS) in recent years has revolutionized our understanding of this kingdom and allowed improved assessment of the diverse range of unculturable fungi that exist, enhancing our understanding of the lung mycobiome in health and CRD [1,7,19,23,24,25,26]. While lung mycobiome research continues to gain increasing traction, various clinical, technical, analytical, and logistical challenges continue to hamper progress in the field [7,8,10,19,27,28,29]. In this review, we will describe the relevance and importance of *Aspergillus*-associated disease and the lung mycobiome in both healthy individuals and those with CRD, with a focus on COPD and bronchiectasis, and address the promise, challenges, and current limitations associated with NGS in understanding fungi and its clinical manifestations in CRD.

## 2. Clinical *Aspergillus* Signatures in COPD and Bronchiectasis

Individuals with chronic respiratory disease, including asthma, COPD, cystic fibrosis (CF), and bronchiectasis, are at clinically significant risk of *Aspergillus*-associated disease [1,5,17,20]. Owing to the anatomical abnormalities that exist and worsen with progressive pulmonary damage observed in these states coupled with the relative and/or absolute immune dysregulation that accompanies disease pathogenesis, clinicians should be aware of important clinical *Aspergillus*-associated signatures that may affect disease morbidity and/or mortality. Furthermore, the high environmental abundance and subsequent routine inhalation of *Aspergillus* spores, the organisms’ immune-evasive capabilities, and the increasing prevalence of azole resistance further challenge clinicians in the management of individuals with chronic respiratory disease [30]. Whether such *Aspergillus*-associated signatures represent genuine ‘treatable traits’ in each respective disease state remains an intense area of ongoing clinical study and research. 

Clinical *Aspergillus* signatures, including the presence of sensitization responses, are established in association with frequent exacerbations in severe asthma while in CF, airway colonization is associated with radiological abnormalities, downregulation of airway vitamin D receptor expression, and enhanced Th2-mediated inflammatory responses, controlled by the *Aspergillus*-secreted virulence factor gliotoxin [4,31,32]. The role of *Aspergillus* in COPD and non-CF bronchiectasis, however, is less clear. Emerging work by our group and others suggests that it has clinical relevance; however, geographic variability must be considered [2,16,21,33,34]. As the isolation and culture of fungi have been problematic in clinical practice, next-generation sequencing (NGS) now provides even greater insight into the ‘unculturable fungal communities’ that exist in health and disease [26,35]. These emerging datasets suggest that the role of fungi extend well beyond *Aspergillus* alone and clinicians need to consider other fungal taxa, including how they may potentially interact with other microbial kingdoms, particularly in the setting of chronically inflamed and diseased airways, such as that observed in COPD and bronchiectasis [9,24]. 

Important work now provides insight into clinical *Aspergillus* signatures relevant for translation and clinical practice in COPD and bronchiectasis. Critically, this work offers an important basis to better understand the role of fungi more broadly in the context of chronic respiratory disease. In COPD, a distinct airway mycobiome profile is observed when assessed against healthy controls, and those who exacerbate frequently further demonstrate enhanced inter-fungal interactions [10]. Interestingly, no measurable effect on the mycobiome profile was identified using longitudinal analyses over the course of COPD exacerbations or in those using long-term corticosteroids; however, unsupervised analysis of mycobiomes in COPD did uncover the important role of *Aspergillus* [10]. While *Saccharomyces* dominance is associated with higher symptomatic burden, the presence of *Aspergillus*, *Curvularia*, and *Penicillium* is linked to frequent exacerbations and higher two-year mortality, suggesting that a key sub-group of individuals with COPD have identifiable high-risk airway mycobiome profiles [10]. Bronchiectasis similarly demonstrates its own distinct airway mycobiome compared to healthy individuals; however, it does appear to be different to the COPD mycobiome, albeit with a consistent presence of *Aspergillus* in this setting, accompanied by *Cryptococcus* and *Clavispora* [8]. Importantly, when *Aspergillus* speciation is considered in stable bronchiectasis, *A. fumigatus* predominates in Asian individuals and *A. terreus* in non-Asian individuals. *A. terreus* is associated with bronchiectasis exacerbations; however, high frequencies of *Aspergillus*-associated disease in general, including sensitization and allergic bronchopulmonary aspergillosis (ABPA), each accompanied by their own mycobiome profile, are evident in bronchiectasis [8].

COPD and bronchiectasis represent respiratory diseases that demonstrate a high prevalence of sensitization, and sub-groups of patients with fungal sensitization are described, suggesting that ‘fungal-sensitized’ disease in these settings may represent an important ‘treatable trait’ [36,37]. This is of significance in bronchiectasis, where inherent patient heterogeneity has plagued clinical trial success and the need for treatable traits of clinical importance [38]. In COPD, fungal sensitization is associated with frequent exacerbations and outdoor and indoor sources of exposure appear important, while in bronchiectasis, ‘immuno-allertypes’ of disease are described, one fungal-driven and characterized by poorer clinical outcomes and a proinflammatory airway milieu [36,37]. While the involvement of basophil biomarkers in the sensitization response to *Aspergillus* in CF has been examined, comparable data is lacking in COPD and bronchiectasis [39,40]. The study of human chitinase activity, chitotriosidase (CHIT1) and acidic mammalian chitinase (AMC), established in CF, has, however, been evaluated in bronchiectasis and demonstrates a key association with individuals who frequently exacerbate in South-East Asian settings [41,42]. However, further work elucidating specific mechanisms for these findings and prospective clinical studies evaluating the usefulness of this potential marker remain to be pursued. 

While our clinical, microbiological, and molecular understanding of *Aspergillus* signatures in COPD and bronchiectasis have advanced in recent years, an appreciation of the ‘overlap’ between the two conditions provides additional challenges [6,19,43,44]. In a prospective, observational, and cross-sectional study of individuals with COPD, bronchiectasis, and bronchiectasis-COPD overlap (BCO), those in the latter group demonstrated the highest frequency and clinical severity of ABPA compared to individuals with an absence of overlap [45]. BCO-associated ABPA is linked with an overall poorer clinical outcome and appears to be of highest risk in individuals with severe bronchiectasis (i.e., with a bronchiectasis severity index >9) [45]. While clearly enforcing further links between COPD, bronchiectasis, and the varying *Aspergillus* endophenotypes and clinical signatures in these disease states, this work is important but requires further assessment of the underlying immune and mycobiome-related mechanisms [25,46]. More broadly, however, collectively, the existing evidence base does serve to confirm the clinical relevance and importance of *Aspergillus*-associated signatures in COPD and bronchiectasis and the urgent need to harness these signatures for improved clinical outcomes. Achieving this necessitates an improved awareness through education, accessibility through testing, and fresh approaches from a research perspective, such as that offered by novel analytics and inter-kingdom analyses [9,12,47,48].

## 3. The Challenges of Sequencing the Mycobiome

Current methods for detecting fungal infection include culture, microscopic examination, serology, and histopathology [5,26,49,50]. However, these methods are challenged by various limitations, including the lack of sensitivity and specificity, time required, sample acquisition, and quality, and may be confounded by the host immunology and prior use of antibiotics and antifungals [5,49,51,52,53,54,55,56]. NGS has been developed to allow for increased specificity and sensitivity [5,26,55,57,58,59]. Although the last decade has seen exponential increases in the use of NGS, its widespread implementation as a routine diagnostic tool remains limited and currently mainly restricted to research settings [60].

Targeted amplicon sequencing utilizes a specific primer to amplify the internal transcribed spacer (ITS) region, the universal DNA barcode for fungi, followed by sequencing [61,62,63,64,65]. This approach permits the identification of diverse fungal taxa, which remained unculturable using traditional methods. Primer selection, however, is a crucial step in targeted amplicon sequencing, influencing taxonomic detection, resolution, and accuracy [26]. As the ITS region ranges in size from between 450 and 700 bp, shorter sub-regions of ITS1 and/or ITS2 are often selected for amplification and sequencing [61,66]. Various studies have compared the accuracy of ITS1 versus ITS2 primer, with inconsistent results. ITS1 primers have been shown to have greater coverage for Basidiomyces while ITS2 demonstrated superior amplification of Ascomycetes [66]. Ali et al. compared primers targeting the ITS1 and ITS2 regions, and found that the ITS2 primer showed better accuracy in predicting mock communities consisting of *Aspergillus*, *Candida*, *Curvularia*, and *Schizophyllum* in comparison to the ITS1 primer [67]. ITS 2 was proposed to have less taxonomic bias, lower length variation, and better detection of lower abundance taxa [26,61,68]. It is, therefore, critical to note that variation in primer bias, amplification bias, and accuracy has been observed and characterized at varying taxonomic levels for primers targeting the ITS1 and ITS2 sub-regions and remains a limitation in assessing mycobiomes using targeted amplicon approaches [61,67,69,70,71].

In contrast, shotgun metagenomic sequencing (mNGS) alternatively provides an unbiased assessment of the total genomic DNA without the need for prior polymerase chain reaction (PCR) amplification of the targeted ITS regions [72,73,74,75]. In addition to unbiased and comprehensive detection of microbes, including fungi, bacteria, and DNA viruses, it provides additional information, including functional profiling and resistance gene determinants. While mNGS clearly holds promise in the detection of fungi, its use in respiratory specimens remains limited [26,76]. Furthermore, the overall low abundance of fungi relative to the overall microbial community and high host genomic content detected by mNGS poses significant additional challenges to the use of metagenomic sequencing for mycobiome analysis. Overcoming such limitations necessitates a reversion to targeted amplicon sequencing approaches as described above [77,78,79]. 

While sequencing can provide a detailed taxonomic and functional composition of microbial communities in the airway, various challenges continue to plague progress in the field (Table 1) [19,67]. Fungi are bound by a rigid cell wall, which requires pre-processing of specimens with mechanical and/or enzymatic lysis prior to DNA extraction [80,81,82]. This process may inadvertently degrade the DNA, adversely affecting the overall yield and quality of downstream sequencing [83,84]. In addition, the low fungal biomass in respiratory samples further compounds the challenges in yielding adequate fungal DNA [84,85,86]. Notably, variations in the fungal DNA yield amongst different commercial kits and extraction methods are now also being increasingly reported [83,87,88].

Contamination and the reproducibility of mycobiome data remain a major issue in the use of NGS for mycobiome analysis [89]. The high sensitivity of NGS coupled with the significant abundance of fungi in the environment results in increased occurrences of potential contamination. To address this, additional steps are required to minimize such contamination, and the incorporation of strong and numerous negative controls during sample acquisition, processing, and sequencing (to identify potential contaminants) is important. Poor reproducibility is a further concern. Inter-individual variability in the mycobiome composition is often reported, despite the lack of replicate samples assessed during the sequencing process. Indeed, prior studies have reported that repeat DNA extraction and sequencing of technical replicates can illustrate a high abundance of specific taxa in one sample but its absence in another, a feature not observed with concurrent bacterial profiling [85]. These data suggest that further optimization of mycobiome sequencing protocols, including technical replicates, is required.

Several sequencing platforms are now available, ranging from short-read or second-generation sequencing (e.g., Ion Torrent and Illumina platforms) to long-read or third-generation sequencing approaches (e.g., Pacific Biosciences (PacBio) and Oxford Nanopore Technologies) [60,90]. Short-read sequencers, particularly the Illumina platform, are widely used globally and have higher base calling accuracy when compared to long-read sequencers [91]. In addition, the Illumina platform is supported by a range of analytical tools and pipelines, making it more favorable for mycobiome studies [90]. Conversely, long-read sequencing approaches generally employ a single-molecule approach to generate the long read length and improve de novo genome assembly [60,90]. While it has lower read accuracy compared to second-generation sequencing, the MinION portable device provides real-time sequencing without space restriction, making it readily accessible to bedside use, particularly during acute infection outbreaks [60,91].

Bioinformatics analyses for mycobiome sequencing remain an underdeveloped area of research, lacking consensus on best practices, including the absence of broadly applicable analytical pipelines [67]. Poorly curated fungal reference databases have consequently affected the accuracy of taxonomic assignment, as large numbers of unidentified operational taxonomic units (OTUs) persist, and poor species-level classification is achieved [61,79,92,93,94]. On the other hand, metagenomic sequencing analysis requires more complex bioinformatic pipelines and expertise in addition to high-performance computing clusters, hindering widespread adoption and application [19,26,79,95]. 

## 4. The Pulmonary Mycobiome in Health, COPD, and Bronchiectasis

### 4.1. The Pulmonary Mycobiome in Healthy Individuals

Advances in sequencing technologies have now clearly established that the bronchial tree is not sterile and contains diverse range of microorganisms even in healthy individuals [96]. Microorganisms play important roles in immune maturation, mucosal barrier function, and the maintenance of homeostasis in the lung microenvironment [24]. Consequently, changes in microbial communities and/or their functional abilities are associated with CRD [26]. The bacterial microbiome has been widely examined and specific microbiome “fingerprints” have been described in relation to CRDs [33]. The role of the mycobiome, however, is less well established, largely due to the inherent methodological challenges associated with mycobiome sequencing as previously described [26,84]. Even fewer studies have evaluated the respiratory mycobiome in health, although high inter-individual variability must be considered in evaluating such datasets due to variation in sample collection and sequencing methodologies, geographic and climatic factors, and individual host exposure between participants in the various study cohorts [35,84,97,98,99,100,101]. While differences in mycobiome taxa exist between studies, the dominant genera in oral, sputum, and bronchoalveolar lavage (BAL) samples include *Aspergillus*, *Cladosporium*, *Candida*, *Mycosphaerella,* and *Malassezia*, with an overall lower abundance detected in BAL [35,97,98,99,100,101]. In a prospective study of healthy Asians, discrepancies in the previously reported dominant taxa were observed and *Candida*, *Saccharomyces*, *Ganoderma*, and *Grammothele* reported [8,10]. In this latter work, ‘paired’ sputum samples were importantly obtained from healthy first-degree relatives that partially (but not fully) controlled for genetic influences but further illustrated important changes in the healthy mycobiome composition with age, where an increased abundance of *Candida* was detectable in older compared to younger ‘paired’ individuals [35]. Taken together, these data suggest the presence of a healthy airway mycobiome, with inter-individual variability likely largely influenced by geographic location and age.

### 4.2. COPD

Microorganisms are often detected in the airways of patients with stable COPD or during acute exacerbations [2]. It is hypothesized that an initial insult (by noxious or infectious stimuli) followed by impaired innate immune defenses and subsequent microbial colonization lead to epithelial injury and inflammation, which perpetuates vicious cycles of chronic infection and airway damage in COPD [102]. While the implications of bacterial and viral infections in COPD are well established, the role of fungi remains unclear despite their high abundance in the outdoor and indoor environments that patients are exposed to [18,36]. This lack of understanding of the role of fungi in COPD is in a large part attributable to the lack of routine fungal testing of airway specimens in COPD and the relatively poor sensitivity of fungal detection methods [26,67]. Patients with COPD demonstrate mucociliary clearance abnormalities and defective immune responses, which, when coupled with long-term inhaled corticosteroid use and frequent bursts of oral corticosteroids (during exacerbations), theoretically predispose patients to fungal persistence in the COPD airway [10]. Indeed, positive fungal cultures from sputum have been associated with increased airway neutrophils and are detected in up to half of COPD patients in reported studies, with the majority representing *Aspergillus* species [2,103,104,105]. 

**Figure 1:**
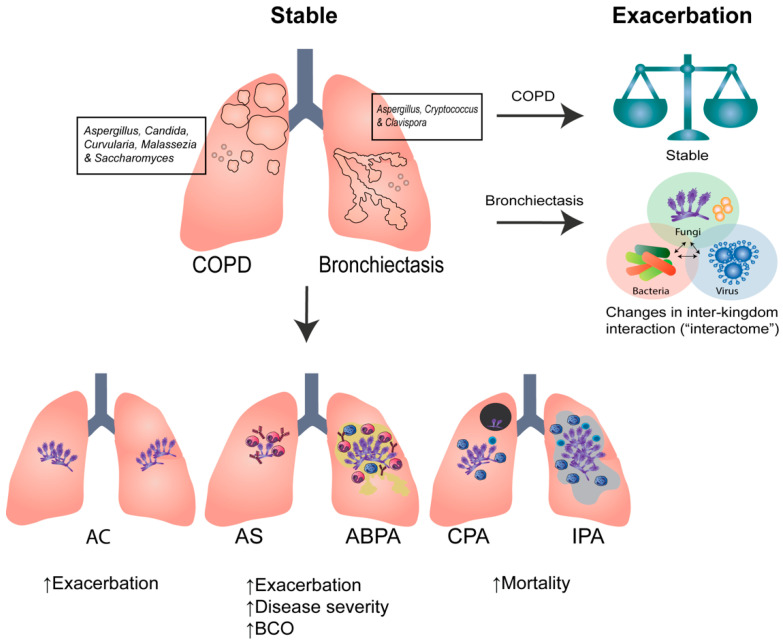
Summary of the pulmonary mycobiome during the stable state and exacerbations in COPD and bronchiectasis, including their association with *Aspergillus*-associated conditions and their clinical consequence. AC: *Aspergillus* colonization, AS: *Aspergillus* sensitization, ABPA: allergic bronchopulmonary aspergillosis, CPA: chronic pulmonary aspergillosis, IPA: invasive pulmonary aspergillosis, BCO: bronchiectasis-COPD overlap, COPD: chronic obstructive pulmonary disease, ↑: increased.

Using targeted amplicon sequencing, distinct fungal mycobiome profiles have been identified in individuals with COPD [5,19,26]. A predominance of *Pneumocystis spp.* is described in COPD with co-existing HIV while *Candida*, *Malassezia*, and *Sarocladium* (Figure 1) have been detected in stable COPD using BAL [27,106]. The dominant fungal organisms remain similar between oral, sputum, and BAL specimens; however, variations are noted in the taxonomic composition. Furthermore, underlying airway inflammatory phenotypes have also been associated with specific mycobiome profiles, with increased *Aspergillus* observed in eosinophilic and *Papiliotrema* in non-eosinophilic COPD [107]. Interestingly, however, comparable mycobiome profiles are observed in eosinophilic and non-eosinophilic asthma [107]. Current mycobiome studies in COPD remain cross-sectional, with limited information available longitudinally. To better understand the stability of the mycobiome in the COPD airway, our group reported a large multicenter longitudinal study that evaluated the sputum mycobiome in COPD during the stable state, during an acute exacerbation, and in the post-exacerbation state [10]. In stable COPD, the airway was dominated by *Candida*, *Saccharomyces*, *Curvularia*, and *Aspergillus* (Figure 1)*,* and significant differences in the mycobiome composition were identified between individuals with COPD from different geographic regions. For instance, individuals from Singapore and Malaysia demonstrated different mycobiome profiles to individuals living in the United Kingdom [10]. Interestingly, the mycobiome remains stable over longitudinal assessment, including analysis during times of stability, acute exacerbation, and 2 weeks post-exacerbation following treatment with oral antibiotics and corticosteroids. Importantly, a lower alpha-diversity during acute exacerbations of COPD (AECOPD) was predictive of mortality at the two-year follow-up. When the mycobiome was compared between COPD patients with or without inhaled corticosteroid use, no significant differences in the airway mycobiome composition and/or diversity were observed [10]. In addition, a “high risk” mycobiome profile characterized by *Aspergillus*, *Curvularia*, and *Penicillium* was identified in association with higher mortality and frequent exacerbations, suggestive of the importance of assessing mycobiomes in COPD [10]. In a subset of patients, a positive correlation between the “high-risk” mycobiome profiles and a sensitization response to the same fungi was identified, suggesting that isolation of a specific fungus or fungal group from the airway can be linked to measurable systemic host immune responses. In a separate study, sensitization to environmental fungi was also found to be prevalent in COPD and associated with increased exacerbations [36]. Metagenomic sequencing of indoor air from patients’ bedrooms further demonstrated a high proportion of fungal allergens, which was correlated with worse symptoms and poorer lung function [36]. Collectively, these findings support the presence of specific airway fungi in COPD that differ from healthy individuals, with important and clinically relevant prognostic implications. The role of geographic variation in the types of fungi, and the contribution of fungal environmental exposure remain areas of interest that require additional study. Importantly, the isolation of fungi from the airway in stable COPD may serve as an important precursor of subsequent sensitization and/or worsening disease and should be interpreted cautiously. This contrasts with bacteriome studies in COPD, revealing that bacteria and fungi likely play different roles in COPD pathogenesis and progression. Rather than assessing a single organism and/or kingdom, future studies evaluating interactions between bacteria and fungi with and without exposure to corticosteroids may provide an improved understanding of their role in COPD.

### 4.3. Bronchiectasis

Bronchiectasis is characterized by a progressive irreversible and pathological dilatation of the bronchi associated with impaired mucociliary clearance, leading to an increased susceptibility to chronic airway colonization and infection and its associated inflammation [1,108,109]. Microorganisms, such as *P. aeruginosa, H. influenzae*, and non-tuberculous mycobacteria (NTM), are all commonly isolated in airways with bronchiectasis [24,108,110,111]. Fungi, notably *Aspergillus,* are increasingly being recognized as potential pathogens in both cystic fibrosis (CF) and non-CF bronchiectasis with an association with airway inflammation and disease severity [5,6,8,112,113,114,115,116]. Impaired mucociliary clearance and mucus-congested airways present an ideal environment for fungal colonization and sporulation. Chitin and β-glucan within fungal cell walls degrade the tissue matrix and further induce pulmonary inflammation while proteases and toxins, such as gliotoxin, promote cytokine production and ciliostatic effects [30,31,117,118,119,120]. *A. fumigatus* conidia evade phagocytic defenses and replicate in the airway, leading to further inflammation and damage [121,122,123]. Such complex airway interactions between the host and fungi induce airway remodeling, propagating further destruction and worsening bronchiectasis [5,6]. While traditional fungal culture and microscopy remain in routine use, they have more recently been superseded by molecular biology (qPCR) and NGS approaches [124]. Next-generation sequencing allows for a more comprehensive profiling of the airway microbiome in bronchiectasis [25,43]. *Pseudomonas*- and *Haemophilus*-dominated microbiomes have been associated with neutrophilic inflammation, and positive correlations have been detected between the bacteriome diversity and lung function in bronchiectasis [125,126,127]. Compared to the bacteriome, the mycobiome has received lesser attention, largely due to limitations and challenges in the application of sequencing methodologies. Nevertheless, mycobiome sequencing, performed by our group and others, has revealed the important presence of a distinct airway mycobiome in bronchiectasis.

The landmark cross-sectional Cohort of Asian and Matched European Bronchiectasis (CAMEB) study utilized a ‘matched’ bronchiectasis cohort from Scotland, the UK, and Singapore/Malaysia to evaluate the airway mycobiome using high-throughput sequencing of the ITS1-ITS2 regions. The bronchiectasis airway was characterized by *Aspergillus*, *Cryptococcus*, and *Clavispora* (Figure 1) [8]. While *Candida* remained the overall dominant genera, interestingly, it was equally detected in the airway of healthy and bronchiectasis cohorts. *A. fumigatus* and *A. terreus* remained the predominant *Aspergillus* species detected in the bronchiectasis airway, with some variation observed between geographic areas but was uniformly associated with frequent exacerbations. A higher *A. terreus* conidial burden was detected in Scotland whilst *A. fumigatus* dominated in the Asian (Singapore/Malaysia) cohort [8]. In addition, increased systemic chitotriosidase activity, a fungal marker, was associated with an increased presence of sputum *Aspergillus* and frequent exacerbations in Asian but not European-based bronchiectasis, further supportive of a potential involvement of *Aspergillus* in bronchiectasis progression [42]. Further assessment of *Aspergillus*-associated disease in bronchiectasis revealed an increased frequency of sensitization and sABPA in bronchiectasis, each with their own distinctive mycobiome profiles and increasing amounts of airway *Aspergillus* [8]. Moreover, sABPA is associated with a higher exacerbation frequency and greater disease severity [8,45]. Fungal sensitization was further assessed in the CAMEB study, and, interestingly, two distinct ‘immuno-allertypes’ were identified: one dominated by sensitization to house dust mite and increased airway chemokines, and a second characterized by fungal sensitization and increased proinflammatory cytokines, with the latter group being associated with greater disease severity [37]. Taken together, these studies support the importance of *Aspergillus* and its associated disease states in bronchiectasis, potentially contributing to disease progression. *Candida* is another commonly isolated fungi from the respiratory tract and an important component of the bronchiectasis mycobiome; however, its equivalent detection in healthy airways makes its clinical significance uncertain [116]. Some studies have indeed found associations between *Candida* and a higher exacerbation frequency; however, these may potentially be attributed to higher antibiotic use leading to the increased *Candida* airway abundance [116,128].

Bacterial and fungal sequencing have each demonstrated importance in bronchiectasis; however, studies are often performed in isolation, addressing a single kingdom, with little known about potential inter-kingdom interactions. To further assess this, our group recently performed the first integrative microbiomics study, which combined concurrent assessment of bacteria, fungi, and viral communities in bronchiectasis [9]. Rather than changes in individual single-kingdom microbiomes, a significant change in the microbial interactions (or the “interactome”) in frequently exacerbating bronchiectasis was detected (Figure 1) [9]. This multi-biome study, therefore, proposes that single-kingdom approaches are insufficient for providing adequate insight into the microbial ecology of the airway in bronchiectasis, further emphasizing the importance of microbial interactions during exacerbations and following antibiotic treatment. 

## 5. Future Directions

Whilst our understanding of the role of the lung mycobiome in healthy individuals and CRDs has improved significantly in recent years, it continues to evolve. Addressing the known limitations of mycobiome studies remains a critical aspect to attaining further progress in this emerging field. Deriving a consensus and best practice methodologies from sample processing to bioinformatics pipelines remain a priority. Fungal reference databases require curation and expansion to allow greater access to fungal reference genomes, especially at the species level [92]. Some initiatives are underway to address this [92]. Collectively, such limitations affect our ability to conduct accurate cross-comparisons between mycobiome studies that currently exist in the literature. Future studies should not only attempt to curate publicly available datasets of mycobiome and fungal genomes but also review the methodologies employed and subsequent results using a ‘meta-analysis’-type approach. Such efforts will allow a further understanding of the true effects from technical variations. 

In addition, understanding inter-kingdom interactions and potential microbial ‘cross-talk’ between microbes in the same and even between organ systems in conjunction with host immune and inflammatory responses will be essential to delineate potential pathways in CRDs that may contribute to disease progression in COPD and bronchiectasis. Dysbiosis of the gut mycobiome with antibiotic or antifungal therapies and the experimental enrichment of *Candida* and *Wallemia* species have already been shown to enhance allergic airways disease with Th2 inflammation, eosinophilic infiltration, and increased airway hyperresponsiveness to house dust mite and *Aspergillus* spores in animal models [129,130,131]. Importantly, these works illustrate the clear presence and relevance of complex interactions between microbes, their virulence, and the host systemic immune response, with far-ranging implications beyond dysbiosis. Future studies assessing mycobiome interactions between various organ systems, including the ‘gut–lung axis’ and inter-kingdom interactions with host inflammatory and immune responses, will provide greater clarity of the precise and potentially translational and/or targetable role for the mycobiome in COPD and bronchiectasis. 

## 6. Conclusions

Fungi remain important pathogens in COPD and bronchiectasis. The role of *Aspergillus* species and the wider lung mycobiome in these disease states represents an emerging field of growing importance, demonstrating associations with poorer clinical outcomes, and some with prognostic implications. While lung mycobiome studies continue to receive significant interest, it remains lesser studied compared to the bacteriome. Progress in the mycobiome field is hindered by various challenges and limitations, which, if overcome, will provide immense potential for greater resolution of our understanding of the role of the mycobiome and its wider clinical utility for respiratory medicine.

## Figures and Tables

**Table 1:** Challenges in next-generation sequencing of the mycobiome.

Stage	Challenges
**Sample processing**	Contamination DNA degradation with lysis of the fungal cell walls Variation in the DNA yield and quality between different commercial kits and extraction methods
**Targeted amplicon sequencing**	Primer bias Amplification bias Target accuracy Data reproducibility
**Shotgun metagenomic sequencing**	Low overall fungal abundance relative to bacteria High levels of host DNA impedes fungal detection High costs Data reproducibility
**Bioinformatics analyses**	Lack of consensus on best practices Limited established bioinformatics pipelines Poorly curated fungal reference databases Large numbers of unidentified taxa Poor species-level resolution

## Data Availability

Not applicable.
